# Assessing the presence of oligoclonal IgM bands as a prognostic biomarker of cognitive decline in the early stages of multiple sclerosis

**DOI:** 10.1002/brb3.2405

**Published:** 2021-11-18

**Authors:** Clàudia Coll‐Martinez, Ester Quintana, Judit Salavedra‐Pont, Maria Buxó, Marina González‐del‐Rio, Immaculada Gómez, María Muñoz‐San Martín, Luisa María Villar, Gary Álvarez‐Bravo, René Robles‐Cedeño, Lluís Ramió‐Torrentà, Jordi Gich

**Affiliations:** ^1^ Girona Neuroimmumology and Multiple Sclerosis Unit, Neurology Department Dr. Josep Trueta University Hospital and Santa Caterina Hospital Girona/Salt Spain; ^2^ Neurodegeneration and Neuroimflammation Research Group, Institut d'Investigació Biomèdica de Girona Dr. Josep Trueta Salt Spain; ^3^ Medical Sciences Department University of Girona Girona Spain; ^4^ REEM, Multiple Sclerosis Spanish Network Instituo de Salud Carlos III Madrid Spain; ^5^ Institut d'Investigació Biomèdica de Girona Dr. Josep Trueta Salt Spain; ^6^ Immunology Department Ramon y Cajal University Hospital, IRYCIS Madrid Spain

**Keywords:** biomarkers, cognitive impairment, multiple sclerosis, neuropsychology, oligoclonal bands

## Abstract

**Background:**

An association has been found between the presence of lipid‐specific oligoclonal IgM bands (LS‐OCMB) in cerebrospinal fluid and a more severe clinical multiple sclerosis course.

**Objective:**

To investigate lipid‐specific oligoclonal IgM bands as a prognostic biomarker of cognitive impairment in the early stages of multiple sclerosis.

**Methods:**

Forty‐four patients underwent neuropsychological assessment at baseline and 4 years. Cognitive performance at follow‐up was compared adjusting by age, education, anxiety–depression, and baseline performance.

**Results:**

LS‐OCMB+ patients only performed worse for Long‐Term Storage in the Selective Reminding Test (*p *= .018).

**Conclusion:**

There are no remarkable cognitive differences between LS‐OCMB– and LS‐OCMB+ patients in the early stages of MS.

## Abstract 2

Although an association has been reported between the presence of lipid‐specific oligoclonal IgM bands (LS‐OCMB) in cerebrospinal fluid and a more severe clinical multiple sclerosis course, no remarkable cognitive differences were found in this study between LS‐OCMB– and LS‐OCMB+ patients in the early stages of MS.

## 1 INTRODUCTION

Multiple Sclerosis (MS) is a chronic inflammatory demyelinating and neurodegenerative disease of the central nervous system (CNS). It is the most common nontraumatic cause of neurological disability in young adults (Grzegorski & Losy, 2017).

Cognitive impairment (CI) is present in between 40% and 65% of MS patients (Ruano et al., 2017). It can appear at the beginning of the disease (Campbell et al., 2017; Chiaravalloti & DeLuca, 2008) and its severity varies depending on the stage of the disease (Ruano et al., 2017). Cognitive deficits have a major impact on patients’ daily functioning, their capability to work, and their quality of life (Campbell et al., 2017; Chiaravalloti & DeLuca, 2008). Even though MS is characterized by a high clinical heterogeneity the neuropsychological profile is relatively well established. Cognitive manifestations of MS mainly include complex attention, information processing speed, episodic memory, working memory, and executive function impairments (Chiaravalloti & DeLuca, 2008; Grzegorski & Losy, 2017; Sumowski et al., 2018).

Given the high prevalence and also the great impact on daily activities of CI it is important to identify risk prognosis factors in order to consider more active clinical and therapeutic actions from the onset of the disease. Certain demographic and clinical data have been identified as predictors of the disease clinical course (age, physical disability, disease subtype, number of relapses in the first 2 years, etc.) (Langer‐Gould et al., 2006), but the evidence is controversial (DeLuca et al., 2015; Ruano et al., 2017) and for some of them such as number of relapses in the first 2 years, the time required is too long for prognostic purposes (Thangarajh et al., 2008). Some neuroimaging factors have been reported (e.g., lesion load and brain atrophy) as potential biomarkers (DeLuca et al., 2015; Eijlers et al., 2018); however, even though these findings are valuable in helping to explain the mechanisms underlying cognitive impairment, they have proved of little clinical applicability for predicting this impairment to date. Besides, some biomarkers present in the cerebrospinal fluid (CSF) have been investigated. Neurofilament light chain (NfL) is one of the most studied biomarkers in a variety of neurological disorders, its concentration in the CSF reflects the ongoing pathology driving to axonal damage (Gaetani, Blennow et al., 2019). In MS NfL has been related to overall cognitive impairment (Gaetani, Salvadori et al., 2019; Kalatha et al., 2019) and also with specific cognitive domain impairment, mainly information processing speed (Kalatha et al., 2019; Modvig et al., 2015; Quintana et al., 2018). Chitinase 3‐like 1 protein (CHI3L1) has as well been investigated as a potential biomarker for cognitive impairment and it has been negatively correlated with information processing speed (Modvig et al., 2015; Quintana et al., 2018). Current findings indicate correlations between both NfL and CHI3L1 levels and cognitive performance measured at the same moment but evidence about their value for predicting cognitive outcomes at middle or long term is scarce. Identification of new and useful biomarkers to predict cognitive impairment in MS patients is desirable.

Local production of antibodies has been described in MS patients, and some have been identified as biomarkers present in CSF (Thangarajh et al., 2008). Since oligoclonal IgG bands (OCGB) are present in around 95% of MS patients (Villar et al., 2010) its presence is widely accepted as a diagnostic biomarker. Some authors have considered OCGB as a prognostic tool for the disease (Gaetani et al., 2021). Regarding cognitive impairment, few studies report worse cognitive performance of those patients showing OCGB+ compared to those OCGB‐ and healthy controls (Anagnostouli et al., 2015; Farina et al., 2017).

Approximately 40% of MS patients also show oligoclonal IgM bands (OCMB) in CSF (Villar et al., 2010), which has been reported as being a risk factor for poor prognosis both clinically and imaging (Perini et al., 2006; Pfuhl et al., 2019; Thangarajh et al., 2008; Villar et al., 2003). Most of those patients (70%) show lipid‐specific OCMB (LS‐OCMB), which are directed against myelin lipids (Beltrán et al., 2012) and are more strongly related to an aggressive disease course than those that are not lipid‐specific (Magraner et al., 2012; Thangarajh et al., 2008; Villar et al., 2003, 2008). The presence of LS‐OCMB has been related to early conversion from clinically isolated syndrome to relapsing‐remitting MS and to a greater likelihood of developing secondary progressive MS. It has also been linked to a higher number of relapses, leading to greater physical disability, lesion load, and greater brain atrophy (Boscá et al., 2010; Ferraro et al., 2013; Magraner et al., 2012; Pfuhl et al., 2019; Thangarajh et al., 2008; Villar et al., 2005, 2008).

Given that cognitive impairment is a symptom of MS and that the presence of LS‐OCMB in CSF is consistently related to a more aggressive disease course it seems reasonable that LS‐OCMB could also predict poorer cognitive outcomes. To the best of our knowledge, this is the first data to be published about LS‐OCMB and cognitive outcomes.

## 2 OBJECTIVE

The aim of this study is to investigate the LS‐OCMB in CSF as a prognostic biomarker for cognitive impairment in the early stages of MS.

### 2.1 Materials and methods

This prospective longitudinal study was carried out at the Girona Neuroimmumology and Multiple Sclerosis Unit (Catalonia, Spain) after the approval of the Ethics Committee of the Dr. Josep Trueta Hospital (code:2016.042). All participants signed a written consent form.

All patients diagnosed with MS between 2011 and 2015 were proposed to participate in this study and all those who accepted were cognitively assessed at baseline. Patients meeting the inclusion criteria and not meeting the exclusion criteria (being illiterate, having neurological alterations other than MS, history of traumatic brain injury, psychiatric disorder, drug, or alcohol abuse, having received cognitive rehabilitation or having been treated with corticosteroids in the 2 months prior to the cognitive assessment) were finally enrolled. Only those who were assessed at follow‐up, 4 years after diagnosis, were included in the analysis. Demographical (age, sex, education) and clinical data (total number of relapses, physical disability‐measured with expanded disability status scale [EDSS], and disease‐modifying therapy) was collected at baseline and follow‐up. The Brief Repeatable Battery (Sepulcre et al., 2006), the Trail Making Test (Peña‐Casanova et al., 2009), and the Weschler Adult Intelligence Scale III Subtest Letter‐Number Sequencing (Peña‐Casanova et al., 2009) were used for the cognitive assessment. Equivalent versions were used whenever possible to avoid practice effects. The Hospital Anxiety and Depression Scale (Ibáñez & Caro, 1992) was also administered. Serum and CSF IgG and IgM were quantified by nephelometry using an Immage 800 nephelometer (Beckman Coulter). IgG and IgM bands were analyzed by isoelectric focusing (IEF) and immunoblotting. Oligoclonal IgM against myelin lipids detection was performed by IEF and antigen‐specific immunodetection as previously described (Thangarajh et al., 2008).

IBM software SPSS^®^ Statistics v.26 was used to perform statistical analysis. Descriptive analysis and between two group comparison (*t*‐test for normal continuous variables, Mann–Whitney *U* test for nonnormal continuous variables, Fisher's exact test or chi‐square test as appropriate for categorical variables) were performed for demographic and clinical data. Linear regression models were used to evaluate cognitive performance between groups adjusting results by age, educational level, anxiety‐depression and basal cognitive performance, all of them potential confounding variables as they are known to have a significant effect on cognitive performance. Thus, a single linear regression model was performed for each dependent variable (raw score of the cognitive test at follow‐up). Variables included in each model were: group (LS‐OCMB+ vs. LS‐OCMB–), age, educational level, HADS score, and baseline raw score in the appropriate cognitive test. Significance was set at *p *< .05.

## 3 RESULTS

A total of 44 patients were assessed at baseline and at follow‐up: all of them showed IgG OCB+; 16 showed LS‐OCMB+ and 28 LS‐OCMB–. Clinical and demographic data by group are shown in Table 1. In this cohort of patients, mean age was significantly higher in LS‐OCMB– patients than in LS‐OCMB+ (41.7 vs. 32.1 years, *p *= .002). No other clinical and demographic variables were statistically significant.

**TABLE 1:** Clinical and demographical data by group at follow‐up

		LS‐OCMB– (*n* = 28)	LS‐OCMB+ (*n* = 16)	*p* Value
Age^†^		41.71 ± 7.93	32.06 ± 11.00	**.002^a^**
Sex	Male	5 (17.86)	4 (25.00)	.702^b^
	Female	23 (82.14)	12 (75.00)	
Education	Elementary school	9 (32.14)	1 (6.25)	.153^b^
	High school	13 (46.43)	10 (62.50)	
	College	6 (21.43)	5 (31.25)	
Total relapses^††^		2.00 (1.00–3.00)	2.00 (2.00–2.00)	.888^c^
EDSS		2.00 (1.50–3.00)	1.50 (1.00–2.00)	.105^c^
Immunomodulatory therapy^‡^	First‐line	19 (67.86)	11 (68.75)	.640^b^
	Second‐line	5 (17.86)	1 (6.25)	

*Note*: Categorical variables are presented by count and percentage. Continuous variables are presented by mean ± standard deviation or median (1st quartile–3rd quartile).

^a^ *t*‐test.

^b^Fisher's exact test.

^c^Mann–Whitney *U* test.

^†^Age was collected at baseline.

^††^Total relapses refer to total relapses since the disease onset, so all relapses before diagnosis were taken into account.

‡8 individuals (4 LS‐OCMB– and 4 LS‐OCMB+) were enrolled in a clinical trial or were not receiving any disease‐modifying treatment at follow‐up. First‐line therapies: interferon‐beta, glatiramer acetate, teriflunomide, dimethyl fumarate. Second‐line therapies: natalizumab, fingolimod, ocrelizumab, cladribine, alemtuzumab.

Data from all linear regression models performed is shown in Table 2, only data corresponding to the variable “group” is shown. No significant differences between groups were observed in cognitive performance at follow‐up except for the Selective Reminding Test—Long‐Term Storage score in which the performance of LS‐OCMB+ patients was significantly lower (β = −9.14, 95%CI −16.61 to −1.68; *p *= .018).

**TABLE 2:** Cognitive performance at follow‐up LS‐OCMB+ vs. LS‐OCMB–

	β‐coefficient	95% CI	*p* Value
LNS	0.997	(−0.667, 2.661)	.231
TMT A	−0.087	(−10.526,10.352)	.987
TMT B	−4.832	(−21.915, 12.251)	.569
BNT	0.401	(−1.963, 2.765)	.733
SRT LTS	−9.149	(−16.610, −1.689)	**.018**
SRT CLTR	−6.279	(−15.288, 2.730)	.166
SRT R	−0.406	(−1.642, 0.829)	.509
SpaRT T	−0.307	(−4.224, 3.611)	.875
SpaRT R	0.546	(−1.220, 2.312)	.535
SDMT	2.795	(−4.182, 9.772)	.421
PASAT	1.664	(−5.402, 8.731)	.634
WLG	1.285	(−2.986, 5.502)	.551

*Note*: Single linear regression of each test raw score at follow‐up. Thus, results are adjusted by age, education, basal raw score, and Hospital Anxiety and Depression Scale score.

CI: confidence Interval; LNS: letter‐number sequencing; TMT: Trail Making Test; BNT: Boston Naming Test; SRT LTS: Selective Reminding Test Long‐Term Storage; SRT CLTR: Selective Reminding Test Consistent Long‐Term Retrieval; SRT R: Selective Reminding Test Recall; SpaRT T: Spatial Reminding Test Total; SpaRT R: Spatial Reminding Test Recall; SDMT: Symbol Digit Modalities Test; PASAT: Paced Auditory Serial Addition Test; WLG: Word List Generation.

## 4 DISCUSSION

Efforts to find useful biomarkers related to cognition are emerging due to its clinical relevance and the possibility they offer of moving towards personalized health care. Being able to discriminate those patients at higher risk of developing CI at diagnosis moment would be really relevant. It could allow us to take clinical and therapeutic active measures at the very beginning of the disease in order to prevent CI, or at least delay it as long as possible. OCB could be highly useful for this purpose as well as cost efficient, since they are usually measured at diagnosis moment and they do not change over time.

Some authors have investigated the relationship between OCGB and cognitive impairment, but the available evidence is scarce. Anagnostouli et al. (2015) observed that OCGB+ patients performed significantly worse on visual memory, but no other significant differences were found among the neuropsychological battery administered. Farina et al. (2017) reported that OCGB+ patients have psychical and cognitive poorer outcomes at 10‐year follow‐up. In these two studies, authors have not measured or taken into account OCMB (nor LS‐OCMB). Minding the presumable presence of OCMB+ (and LS‐OCMB+) patients among OCGB+ samples we should consider the possibility of some influence of OCMB on its results; as pointed out by Pfuhl et al. (2019) about OCBG and conversion from CIS to definite MS. On the other hand, when assessing clinical utility of OCGB as a potential cognitive prognostic biomarker, we should consider that its high prevalence (95%) among MS patients (Villar et al., 2010) makes its discriminative power to be limited. OCMB, and particularly LS‐OCMB, has been identified as a prognostic biomarker for poor clinical and radiological evolution (Ferraro et al., 2013; Magraner et al., 2012; Perini et al., 2006; Pfuhl et al., 2019; Thangarajh et al., 2008; Villar et al., 2003).

For all these reasons, we aimed to assess LS‐OCBM as prognostic biomarker for cognition with follow‐up cognitive measures. To our knowledge, this is the first longitudinal study investigating cognitive outcomes related to LS‐OCMB.

No differences were found regarding number of relapses or EDSS in our cohort, unlike findings previously reported. This could be explained by the small cohort size in the present study. A significant difference of age was observed in this population, being LS‐OCMB+ patients younger than LS‐OCMB– patients. This finding is similar to Pfuhl's et al. (2019) data and in contradiction with other previous reports (Magraner et al., 2012; Thangarajh et al., 2008; Villar et al., 2010). It is important to note that Pfuhl et al. (2019) only measured OCMB, not taking into account lipid‐specificity. However, in line with their suggestion, the observed age difference between groups could indicate earlier debut of those LS‐OCMB+ MS patients explained by a higher disease activity driving to earlier symptom developing. Otherwise, this finding might be due to some sampling effect. Our results on cognitive outcomes should not be affected by the observed age difference since we used statistical methods to adjust results by age (and other variables).

Regarding cognition our results do not show LS‐OCMB to have a remarkable impact on cognitive performance after 4 years of follow‐up. Only in a single score of the verbal memory test, which assesses long‐term storage, did we find significant differences. No other differences or tendencies were found in other memory scores (e.g., delayed recall) nor in other cognitive domains. However, our results may indicate incipient differences that are not evident in early stages of the disease: although a 4‐year period is long enough to observe differences in clinical and radiological features, it may be that more time is needed to observe cognitive changes. This might be due to compensatory mechanisms: changes in cerebral activation (Rocca et al., 2015) or active strategies used by patients to improve their performance in tasks (Sumowski et al., 2009), which are especially active in early stages of the disease. Thus CI seems to appear when compensatory mechanisms can no longer compensate brain damage (Sumowski & Leavitt, 2013). The small number of participants and possible practice effects, despite efforts to avoid this phenomenon, are limitations of the study.

Further investigation with a larger cohorts and longer disease duration is needed to clarify whether the presence of LS‐OCMB can predict differences in long‐term cognitive outcomes.

## 5 CONCLUSION

Whereas reports comparing the clinical and radiological evolution of LS‐OCMB+ and LS‐OCMB– MS patients have found significant differences in these respects, the present cognitive study does not find remarkable differences in the cognitive evolution of these two types of patient 4 years after diagnosis. Future studies with a longer follow‐up period and larger cohorts are needed to clarify whether the presence of LS‐OCMB can predict differences in long‐term cognitive outcomes in MS.

## FUNDING

This research received no specific grant from any funding agency in the public, commercial, or not‐for‐profit sectors.

## CONFLICT OF INTEREST

The authors declared no potential conflicts of interest with respect to the research, authorship, and/or publication of this article.

### PEER REVIEW

The peer review history for this article is available at https://publons.com/publon/10.1002/brb3.2405

## Data Availability

Data could be available under request.
